# The context, mechanisms and outcomes of intergenerational programmes involving people living with dementia in scotland: A realist, qualitative study

**DOI:** 10.1177/14713012251317767

**Published:** 2025-02-13

**Authors:** Heather Emond, Fiona Kelly

**Affiliations:** Queen Margaret University, Scotland; Faculty of Social Sciences, 7622 University of Stirling, UK; 3122 Queen Margaret University, Scotland

**Keywords:** intergenerational programmes, dementia studies, realist evaluation, intergenerational, scottish policy

## Abstract

Intergenerational programmes, involving activity-based interventions designed to promote mutually beneficial interactions between participants, have been used in Scotland and further afield as a means of generating social inclusion between different age groups. There is growing interest in the potential outcomes of intergenerational programmes for people living with dementia in particular, with policy in Scotland recognising that people living with dementia and their carers may be at greater risk of loneliness and social isolation. Given this interest, there is a need to explore what ‘intergenerational best practice’ may look like for people living with dementia. Using data from semi-structured interviews with thirteen stakeholders involved in intergenerational practice and/or dementia policymaking, this study explored the contextual factors, mechanisms, and outcomes of intergenerational programmes in the Scottish context. Stakeholders perceived the concerns of carers, perceptions of risk, along with inaccessible venues and transportation to be important contextual factors. Mechanisms that helped ensure programmes offered full and appropriate participation opportunities included ongoing, flexible programme planning; the provision of purpose and roles; and the use of older participants’ preferences, lived experience, and personhood. Overall intergenerational programmes were perceived to have the potential to promote beneficial outcomes for older participants living with dementia in Scotland.

## Introduction

As part of the plan to take forward Scotland’s strategy on tackling social isolation and loneliness, the Scottish Government recommended that community organisations consider training and access support ‘on how to develop and recognise intergenerational best practice, to ensure increased opportunities for good quality interactions between generations’ (Scottish Government, 2023b, p. 19). The original strategy, entitled ‘A Connected Scotland’ and published in 2018, identified ‘intergenerational dialogue’ as being vital in the prevention of exclusion, isolation, and age-based discrimination so that ‘people of all ages are more included in their communities’ (Scottish Government, 2018, p. 48).

Dementia was recognised within the 2018 loneliness strategy as having an impact on loneliness and social isolation, with people living with dementia and their carers being ‘at risk of losing touch with wider family, friends and community connections as a result of the illness’ (Scottish Government, 2018, p. 32). Scotland’s 2023 dementia strategy described how, for many, the lived experience of dementia continued to be lonely, isolated, and affected by stigma, signalling there was still more to be done to make Scottish society dementia-inclusive (Scottish Government, 2023a). There are an estimated 90,000 people living with dementia in Scotland today, including around 3,000 people under the age of 65 (Alzheimer Scotland, 2024b). Given this policy agenda, there is increased interest within Scotland and further afield in terms of how intergenerational approaches may be developed in order to enhance social inclusion and wellbeing for people living with dementia.

## Intergenerational programmes involving people living with dementia

Intergenerational programmes are understood to involve the ‘purposeful bringing together of different generations in mutually beneficial, planned activities’, with such activities designed to increase ‘cooperation, interaction or exchange between the generations via sharing of knowledge, skills, and/or experiences’ (Ayala et al., 2007, p. 47). Intergenerational dementia programmes can be positioned as an activity-based intervention involving people living with dementia and younger age groups that have the aim of promoting intergenerational inclusion alongside a range of other benefits.

Qualitative studies have demonstrated that intergenerational dementia programmes have resulted in the building of connections through play (Hernandez et al., 2022), friendship work (Skropeta et al., 2014), and relationship building between participants of different ages (Canning et al., 2020). Programmes have also generated a sense of home and belonging (Hernandez et al., 2022) and allowed older participants and their carers to be accepted and included, feeling themselves part of a dementia-friendly community (Harris & Caporella, 2019). Interactions between people living with dementia and younger participants have been found to be generally positive (Di Bona et al., 2019), and older participants have indicated that they have benefited from participating in programmes (Jarrott & Bruno, 2007). However, there is evidence that there are elements of intergenerational programmes that people living with dementia do not like, such as noise, commotion, and the children’s impoliteness during programmes (Jarrott & Bruno, 2007). When asked to consider a proposed shared-site programme, Weeks et al. (2020) found that a number of nursing home residents reported concerns such as ambiguity about the role for residents in the programme and the commitment required, the behaviour of children while on-site, and concerns about the safety of the children.

In terms of quantitative research, intergenerational activities involving people living with dementia have been found to achieve higher wellbeing scores (Lokon et al., 2019) and enhanced engagement (Baker et al., 2017; Lee et al., 2007) compared to regular activities. Janke et al. (2019) found that older participants who engaged more constructively and actively in intergenerational sessions exhibited more pleasure and demonstrated more helping behaviours. Moreover, they found that increased levels of pleasure were associated with higher quality of life (Janke et al., 2019). Chung (2009), similarly, reported significant pre- and post-programme differences on measures of Quality of Life in Alzheimer’s disease and the Chinese version of the Geriatric Depression Scale in relation to an intergenerational reminiscence programme. In an observational study, Caspar et al. found that ‘pleasure and general alertness were the two visible emotions observed most often’ during the programme (Caspar et al., 2019, p. 158).

However, several studies have reported mixed findings, such as no differences over time on quality of life, agitation, or sense of community arising from intergenerational sessions (Low et al., 2015), and no change in cognitive status for older adults during their participation in an intergenerational reading programme (Isaki & Harmon, 2014).

## Intergenerational programmes and realist evaluation

This paper reports on research examining how intergenerational programmes involving people living with dementia have ‘worked’ in the Scottish context through a qualitative investigation of stakeholder perspectives. Intergenerational dementia programmes are complex interventions, involving different participant groups as well as different services and organisations. In order to ‘unpick’ this programme complexity, this study adopted a realist evaluation approach. Realist evaluation aims to investigate ‘what works for whom in what circumstances…and why’ and thus assumes that programmes are not only complex but unfold within and across complex social contexts (Pawson, 2013, p. 15).

The realist evaluation lens of programme implementation is structured around the context-mechanism-outcome (CMO) analytical configuration. The context of a programme involves different aspects, such as the characteristics and capacities of the various stakeholders and participants; the interpersonal relations of those involved; the institutional settings local to the programme; and the wider social, economic, and cultural setting of the programme (Pawson, 2013). Contexts are therefore critical towards understanding how programmes work. Mechanisms, moreover, bridge contexts and outcomes by involving the interaction ‘between particular inputs (or resources) and human reasoning, which produces a particular outcome (or not)’ (Wong et al., 2013, p. 1017). Mechanisms are thus fundamental to the development of the programme theory, and they explain why a programme ‘generates particular outcomes in particular contexts’ (Wong et al., 2013, p. 1016). Outcomes are, finally, the patterns of change that occur, or not, following programme implementation. Using this approach, this research sought to examine the following questions:

• What contextual factors have stakeholders in Scotland perceived to be important towards the implementation of intergenerational programmes involving people living with dementia?
• What mechanisms have stakeholders perceived to contribute to how programmes ‘work’, allowing people living with dementia to take advantage of the opportunities and resources created within programmes?
• What outcomes have stakeholders observed arising from intergenerational programmes for people living with dementia in Scotland?

## Methods

Data for this study were collected between 2020–2021. Qualitative semi-structured interviews were undertaken with stakeholders involved in intergenerational practice and/or dementia policymaking in Scotland, with participants possessing relevant expertise gained through their professional experiences. A semi-structured interview method was deemed appropriate due to its ‘versatility’ allowing for the interview structure to be varied according to the needs of the research (Kallio et al., 2016, p. 2955). The interviews required a degree of structure to allow for exploration of the context, mechanisms, and outcomes of intergenerational programmes in Scotland in keeping with the realist evaluation approach adopted for the overall study design (Mukumbang et al., 2020; Pawson, 2013). However, it was important to provide opportunities for participants to bring their own interpretations and meanings through flexibility within the interview process (Galletta, 2013; Kallio et al., 2016).

The Queen Margaret University Business, Enterprise and Management Ethical Approval Committee approved the interviews on November 24, 2020. Respondents gave written consent for review and signature before starting interviews. Consent was further reiterated at the start of every interview. Recruitment was undertaken between December 2020 – April 2021 through a purposeful sampling strategy aimed at selecting individuals who would be knowledgeable about the topic under investigation (Palinkas et al., 2015). Potential participants were identified through internet research in which websites and documents were evaluated to identify key organisations and individuals with relevant experiences. Participants had gained relevant experiences through direct involvement in intergenerational programme implementation, involvement in programme funding and evaluation, and/or participation in networks aimed at promoting intergenerational approaches. Internet research was combined with coordination with intergenerational networks and snowballing, whereby participants were asked if they could recommend additional stakeholders who may wish to be involved.

Thirteen interviews were conducted between January and May 2021. Interviews ranged from 45 minutes to one and a half hours in length. Due to restrictions on meeting in person in Scotland during the COVID-19 pandemic, all interviews were conducted remotely through either Zoom or Microsoft Teams.

## Data analysis

Following transcription and anonymisation, qualitative thematic analysis was undertaken in order to identify, analyse, and report patterns within the data (Braun & Clarke, 2006), and as the basis for theorisation. The thematic analysis of the interview transcripts combined both inductive and deductive elements. Deductive analytical components included organising coded excerpts according to the CMO configuration of realist evaluation to build a theory of how stakeholders had perceived intergenerational programmes to ‘work’ in Scotland. Moreover, a realist synthesis systematic review was undertaken prior to commencing the interview data collection, which involved substantial engagement with previous research undertaken internationally on the topic (Emond, 2023). However, no pre-determined codebook or framework was used when coding the interview data and thus an exploratory, inductive approach was taken.

Data were coded using Braun and Clarke’s guidelines for conducting rigorous thematic analysis (Braun & Clarke, 2006) and were managed through NVivo software version 12.6.0. Content codes were developed and documented within a codebook, with each code assigned a definition, and instructions for use if required. Codes were synthesised into themes and organised according to the categories of Context, Mechanism, and Outcome in order to address the research questions. As an additional step towards achieving close cooperation with stakeholders, member checking was implemented with interview participants throughout autumn 2021 (Nowell et al., 2017). This process involved sharing an overview of the themes with participants and providing them with an opportunity to respond with their comments, thereby allowing them to review the ‘summarized data to see if they accurately [reflected] their intents and meanings’ (Guest et al., 2012, p. 93). One participant requested a second-stage member review in early 2022.

## Findings

### Interview participants

Participants worked within the non-governmental, voluntary sector or local government, and notably the majority were female (85%), which may be related to the higher proportion of women employed in the adult social care workforce in Scotland (Scottish Government, 2022) as well as the higher proportion of women caring for someone living with dementia (Scottish Government, 2023a) (Table 1).

**Table 1.** Characteristics of interview participants.

Characteristics	No. of participants
Gender	
• Female	11 (85%)
• Male	2 (15%)
Sector	
• Non-governmental, voluntary sector	10 (77%)
• Local government	3 (23%)
Job level	
• Manager	6 (46%)
• Coordinator	2 (15%)
• Director	2 (15%)
• Organisational officer	2 (15%)
• Board Member/Trustee	1 (8%)
Region	
• Scotland-wide	3 (23%)
• Central Scotland	2 (15%)
• Lothians	2 (15%)
• Southern Scotland	2 (15%)
• Strathclyde	2 (15%)
• Highlands & Islands	1 (8%)
• UK-wide	1 (8%)
Due to rounding, not all percentages add up to 100	

### Context, Mechanisms and Outcomes

Table 2 summarises findings in relation to the context, mechanisms and outcomes of intergenerational programmes involving people living with dementia in Scotland (Table 2).

**Table 2.** The context, mechanisms and outcomes of intergenerational programmes involving people living with dementia in Scotland.

Contextual factors	• *Settings and diversity of older participants*
	Interviewees perceived a need to implement programmes across whole communities and to involve people living with all stages of dementia where possible
	• *Barriers to participation*
	Potential barriers described by interviewees included the concerns of carers, perceptions of risk, and accessibility issues with venues and transport
Mechanisms generating outcomes	• *Flexible planning*
	Interviewees described the importance of ongoing, flexible planning towards ensuring beneficial outcomes
	• *Purpose and roles*
	Interviewees perceived that providing roles for older participants within programmes could enhance purpose and sharing
	• *Preferences, lived experience and personhood*
	Interviewees described how participant preferences could inform activity development and consolidate voluntary participation, and further recommended trauma-informed approaches and person-centred language
Observed outcomes	• *Forming relationships*
	Interviewees perceived relationships to form between all participants through the social interactions enabled through programmes
	• *Enjoyment*
	Interviewees observed enjoyment among older participants during programmes though recognised that enjoyment could fluctuate through time
	• *Wellbeing*
	Interviewees described positive impacts on the wellbeing of older participants, such as improved appetite and mobility
	• *Engagement*
	Interviewees felt that intergenerational programmes could offer variety within the routines of older participants through inclusive activities that were more engaging

### Context 

#### Settings and diversity of older participants

Interviewees perceived a need to implement intergenerational programmes across whole communities and not just within the provision of services:

(It) involves the whole community, whether it’s the local school, whether it’s the youth club down the road, whether it’s the church you know, or the walking group, you know, it’s about a whole community approach. (Participant 005).

Interviewees described a need to involve people living with all stages of dementia within intergenerational programmes if this coincided with the preferences of individuals:

And early onset right through to end-stage dementia, there’s a need, there is a need. Definitely. (Participant 06).

Moreover, interviewees gave examples of people living with late-stage dementia participating successfully within programmes:

I mean, the one bit of work they were talking about is they had a nursery and they had a lady with really advanced dementia that really was struggling in the community and she started going there and she was very mobile… (Participant 12).

Nevertheless, interviewees also recognised that participation opportunities may be fewer for those whose dementia was more advanced:

There’s not a lot of things out there for people whose dementia is further on, you know… (Participant 02).

#### Barriers to participation

Interviewees described a number of barriers to intergenerational participation for people living with dementia in the Scottish context. Firstly, the perceptions of carers for people living with dementia were seen to be important in terms of organising programmes, with carers occasionally voicing concerns:

…a lot of the carers who didn’t know that we did this would say, have you got children in the unit? And I would be like, yeah, we get them, they come twice a week. And they were like, oh and how does that go? Is that alright? Because a few people were maybe a bit unsure about it, you know… (Participant 03).

Perceptions of risk more broadly were seen to have the potential to make programme implementation more difficult. Perceptions of risk included the idea that people living with dementia are vulnerable and need to be kept safe:

I think there is a bit of a perception of we must treat them with kid gloves and wrap them in cotton wool. And that’s maybe to the detriment of the older people who have dementia, or any older people. (Participant 04).

A second perception centred on the risk that people living with dementia may pose to children and young people as a consequence of their condition:

They’re often not in touch with children or people are fearful of having children be around them because of the unpredictable nature of dementia. (Participant 03).

Venues for activities to take place in, along with transport to and from venues, were also viewed as presenting barriers to the participation of people living with dementia and arguably heightened the perception of risk involved in programmes:

I think…venues is a big one because a lot of the time there is maybe not a lot of choice, especially some of the more rural communities…And sometimes it’s maybe not ideal in terms of its layout or accessibility and there’re things they can do to help that but maybe not sort of make it completely inclusive. (Participant 09).

### Mechanisms

#### Flexible planning

One interviewee recommended that people living with dementia and their carers be involved in programme planning to ensure programmes reflected what they wanted:

I think the important thing for me…is that, when these types of programmes are developed, or pieces of work, that, you know, that there are conversations and a bit of research done ahead of that, so talking to who the beneficiaries of that support might be, what is it they want, what do they want it to look like. (Participant 05).

One interviewee described the flexibility required in planning intergenerational programmes involving people living with dementia in order to best meet their needs on the day:

And it’s just about changing it all the time, so that if something is not working – I don’t mean that you wouldn’t do it again, because you could do the same activity the following week and it would go down a storm, it’s just because people, especially with dementia, it really is depending on how they’re feeling at that moment in time, they’re emotions are very, very acute, you know so when they’re happy, they’re very, very happy but when they’re sad, they’re distraught. (Participant 03).

Another interviewee described the importance of planning activities that would appeal to both older and younger participants:

I suppose it’s again balance…trying to find that balance when you are bringing together people living with dementia and that sort of younger group of people who are not living with dementia, some of the needs are different. So it’s trying to find a good middle ground sometimes to make sure the activity is engaging for both. (Participant 09).

#### Purpose and roles

Interviewees described the importance of providing roles and giving purpose to people living with dementia during intergenerational programmes:

I know from my experience of working with the people with dementia…that certainly older people, people who are experiencing illness, people want to feel that they’ve got a purpose, that they’ve got meaning in life, that they’ve got something that’s worth sharing. (Participant 01).

Several interviewees described how the past careers of older participants formed the basis of generating roles and developing activities, and how this could in turn be beneficial for younger participants:

He brought pictures of him when he was a policeman, and he had a dog and so you know he did a little presentation and he brought these pictures in for the kids and so that was something just from sitting chatting and how excited they were about what he did, you know? (Participant 02).

Intergenerational programmes were perceived to provide roles to all participants that would have otherwise been lacking outside of programmes:

So it worked, well, I would say it worked because there were these people who didn’t have a place or a role and they found a mutual place and role together. (Participant 04).

#### Preferences, lived experience and personhood

The preferences of older participants were described as being used to inform activity development and to ensure participation was voluntary:

Sometimes there was an opt-out or people weren’t forced to do whatever it was, they didn’t have to sit there and do a particular craft, they could get involved in making tea or just sitting, watching, listening, or having a wee wander about. So it’s important to have other options I think for people so they don’t feel coerced into taking part in a group activity if they don’t feel like it that day. (Participant 04).

This may involve recognising that some older people may have a preference not to interact with younger people:

I mean don’t get me wrong, there are people who don’t like children and you have to be very clear on that and you have to be very clear in their care plans… (Participant 03).

It may also involve recognising when an older person’s dementia has progressed to a stage where participation in programmes is no longer advisable:

I think for some groups of people with dementia it can be a really good opportunity…but for other people depending on the stage of dementia they are at, or just depending on them as an individual- because it is about individuals- it might not be as beneficial. (Participant 05).

Intergenerational programmes were, similarly, seen to provide older participants with opportunities to share their lived experiences with others:

I think it also gives people a sense of worth and value, because what they are doing is almost, kind of, transferring their learning to someone else and their experiences to someone else and that makes people feel good and feel worthy, when often at some stages of people’s dementia journeys a lot of that is taken away for different reasons. (Participant 05).

However, interviewees also advised that organisers consider the lived experiences of older participants and how this may impact their participation in programmes:

There’s also a lot of research around trauma and things like that for people as well, so we need to be mindful that some people in their earlier lives might have experienced a degree of trauma, too…So I think that’s why it is so important to really get to know the people that you are going to be working with...(Participant 05).

Interviewees also described the importance of taking a person-centred approach towards ensuring beneficial outcomes through, for example, the language implemented within programmes:

I think just as well something about the language used and people and the roles… well, if people are called ‘patients’ or something like that, it’s obviously going to put a label and a stigma on them, whereas if everyone is just a volunteer or a buddy or something neutral, you know, where people don’t have a label assigned to them because of whatever diagnosis they might have or any other background thing… (Participant 04).

### Outcomes

#### Forming relationships

Relationships were described as a key outcome not just for older participants but for all participants, and were linked to the social interactions enabled through programmes:

They all knew the individual children that came along, so they had built up such a rapport that they actually knew, and this is people with dementia, but they knew all the kids that came along. (Participant 02).

Crucially, intergenerational programmes were seen to create opportunities for relationships that would otherwise be lacking outside of organised activities:

But they’re forming relationships and friendships with people that are maybe something that wouldn’t happen, they might not come into contact with an older person and the older people probably may not come into contact with many younger people, so. (Participant 06).

Relationships were described as the key aim of intergenerational programmes involving people living with dementia:

I can cite one…experience that remains with me, a young boy using his iPad to show an elderly lady how to draw. And the intensity of the relationship in that moment was just priceless, it really was, for both of them. To me, that is the key to good intergenerational engagement. (Participant 13).

Intergenerational programmes were perceived to provide an enabling context for such relationships to develop between participants:

So I think…some of those preconceptions which I think can perhaps hamper interactions in bringing different people together just aren’t there or they disappear almost instantaneously. And there then becomes a real ease, we find, of the coming together. (Participant 11).

#### Enjoyment

Interviewees had observed enjoyment among older participants during intergenerational programmes:

…from the seventy-year-old to the ninety-nine-year-old, they really, really enjoyed having the children there and it really made them, they said it made them feel young again, you know, just to sit with them and listen to their banter and talk and things so. Yeah, they really enjoyed everything and all different aspects of the day. (Participant 02).

The involvement of younger participants, moreover, was perceived to heighten the poignancy of enjoyment for older participants living with dementia:

So it’s about finding something that does make them happy so that they have, they may not remember the task, like so if I had said to a guest the following day what we did the day before, they might not remember the actual task but they will remember the feeling, so they will have the emotional hangover of being happy, you know, that they had a nice time and that there were children involved. (Participant 03).

However, interviewees also recognised the potential for older participants’ enjoyment to be tempered by the progression of their dementia:

Because even people you thought you knew well and who had, the week before…really enjoyed doing a certain activity – because of the progression of their dementia this time they didn’t. (Participant 03).

#### Wellbeing

Interviewees perceived intergenerational programmes to have a positive impact on the wellbeing of older participants living with dementia, such as their appetite and mobility:

And we found that even people ate better because the children were there, and they were all eating. (Participant 03).

Interviewees described how programmes could make older participants feel more positive about their wellbeing:

It can be part of them living well with dementia. (Participant 08).

However, one interviewee did recognise that programme evaluation was difficult and attribution issues could arise particularly in relation to wellbeing improvements, whereby the effects of the activities versus intergenerational components were difficult to untangle:

So we can’t mix it up, so if we say, oh, we’re going to do evaluation on it, and say okay right, what’s happened here? We can’t say that their health has improved because it might have been the exercise that improved their health. (Participant 06).

#### Engagement

Interviewees described intergenerational programmes as a way to make their services more engaging, and to bring variety into the lives of some older participants:

I think a good outcome from some of the work that we have seen is that it just brightens someone’s day to have something different happen that moves away from that mundane day-to-day structure, so I think there’s that, you know, alleviating that social isolation and loneliness, I think that’s a good outcome for intergenerational work. (Participant 05).

The engagement of older participants living with dementia was further observed to be heightened during intergenerational programmes:

Yeah, you know, we really found there was a huge difference in their engagement, you know, people that would normally maybe just sit with a paper or something like that were actually getting up and doing games or playing musical instruments and that was all coming back to them because they were trying to teach the children something that they knew. (Participant 03).

## Discussion

The findings from this study provide valuable insight into how stakeholders have perceived intergenerational programmes involving people living with dementia to have ‘worked’ in the Scottish context. Interviewees described a number of contextual factors in Scotland that they perceived to be important towards the implementation of intergenerational programmes. Interviewees considered there to be a need for intergenerational programmes across a variety of settings involving older people living with all stages of dementia, including advanced dementia. Multiple studies have demonstrated that the involvement of older participants living with more advanced dementia within intergenerational activities is possible (Canning et al., 2020; Di Bona et al., 2019; Janke et al., 2019; Jarrott & Bruno, 2007; Lee et al., 2007; Lokon et al., 2017; Lokon et al., 2019; Skropeta et al., 2014; Zamir et al., 2021).

Caregiver concerns along with general perceptions of risk were perceived to be important contextual factors in Scotland that could present potential participation barriers. Jarrott and Bruno found that a small minority of caregivers of older adults (2 out of 50) ‘specified concerns for their family member’ participating in intergenerational programmes (Jarrott & Bruno, 2007, p. 250). Research undertaken by Weeks et al. in relation to a proposed shared-site intergenerational programme indicated that perceptions of risk could be held by the different groups involved in programme development. Nursing home staff were concerned that ‘the increased presence of children in the nursing home would cause more illness among the residents’ (Weeks et al., 2016, p. 294). Nursing home residents themselves expressed some concerns that children would not be well-behaved and would cause too much commotion in the nursing home (Weeks et al., 2020).

Inaccessible venues and transportation were perceived to be contextual factors that similarly could present participation barriers. Alzheimer Scotland actively promotes the development of dementia-friendly communities in Scottish regions (Alzheimer Scotland, 2024a) and the 2023 dementia strategy for Scotland recognised dementia-friendly communities to be a key pillar of post-diagnostic support (Scottish Government, 2023a). Nevertheless, communities can still be difficult to navigate for people living with dementia. Consequently, the activity-space of people living with dementia may ‘shrink’ over time (Gan et al., 2022, p. 350). This accords with the perception of one Scottish stakeholder who felt that there were fewer opportunities available to people living with advanced dementia in the community.

Understanding how contextual factors differ across programmes implemented in different settings would be beneficial. While risk perceptions in care homes may centre on issues of sickness and infection control (Weeks et al., 2016), within community-based programmes risk may fluctuate depending on the accessibility of the transport and venue used for a programme. A number of studies have focused on developing evidence-based recommendations to enhance the dementia friendliness of different environments (Gan et al., 2022; Handley et al., 2017), and these recommendations could be used to address contextual factors that have the potential to create participation barriers.

Interviewees also identified a number of mechanisms they perceived to have contributed to how intergenerational programmes ‘worked’. Stakeholders emphasised the importance of planning in an ongoing, flexible manner in order to optimise intergenerational programmes and ensure activities appealed to all participants. The type of intergenerational activity implemented within a programme has been found to be less important than the need to ensure that activities themselves are meaningful to all participants (Caspar et al., 2019; Galbraith et al., 2015), avoiding child-orientated environments that may be infantilising for older participants (Lee et al., 2007). Femia et al. stressed the importance of implementing ‘well-conceived’ programmes that ‘planfully’ bring different generations together (Femia et al., 2008, p.285). Gigliotti et al. (2005) similarly described how facilitators in one programme had continually and purposefully worked towards achieving common goals.

To ensure programmes are appropriately planned and implemented, Gigliotti et al. recommended that staff receive ongoing intergenerational training to gain essential skills and knowledge (Gigliotti et al., 2005). Intergenerational best practice can involve a degree of multidisciplinary cross-training encompassing both gerontology and child development (Jarrott & Bruno, 2007). Programmes involving people living with dementia may require more intensive cross-training incorporating, for example, staff training in dementia care centred around managing behaviour and ensuring person-centred approaches (Spector et al., 2016).

The provision of roles that culminate in a sense of purpose for older participants was also identified as a key mechanism by interviewees. Intergenerational programmes have often provided opportunities for people living with dementia to assume a mentoring or teaching role in their interactions with younger participants (George, 2011; Isaki & Harmon, 2014; Lee et al., 2007). There is also evidence to suggest that younger participants can learn about different careers through interacting with older participants and hearing about their life histories (Biggs & Knox, 2014). Moreover, Weeks et al. found that continuing in social roles was of importance to older adults, particularly those living in care facilities, whereas role ambiguity during intergenerational programmes could be a key concern for older participants (Weeks et al., 2020).

Using the preferences of older participants in order to develop and enhance intergenerational programmes was another key mechanism perceived by interviewees. Janke et al. recommended that recreational therapists use assessments with older participants along with observations during activities to ensure that people living with dementia are benefitting from their participation and have a desire to continue engaging (Janke et al., 2019). Lee et al. advocated that Montessori-based programming be used alongside updated data gathering as a way to ‘accommodate changes in cognitive and physical functioning in both older adults and children’ (Lee et al., 2007, p. 482). Hernandez et al. recommended that service providers ensure that participants have choice and control over their attendance in recognition of the fact that intergenerational programming would not be suitable for all (Hernandez et al., 2022). Moreover, concepts such as personhood and person-centred care have informed intergenerational programmes and have been found to be important in the relationship building process between participants (Lokon et al., 2012).

Interviewees observed several outcomes arising for people living with dementia as a result of their participation in intergenerational programmes, including relationship building along with outcomes related to engagement and wellbeing. The finding from the Scottish context that intergenerational programmes result in relationship building between older and younger participants is supported by a number of studies (Canning et al., 2020; Gigliotti et al., 2005; Jenkins et al., 2021 ). There is also evidence in the literature that intergenerational programmes are enjoyable for people living with dementia. In relation to the ‘Grandfriends’ intergenerational programme, Low et al. found that enjoyment was significantly higher during the Grandfriends sessions compared to usual activities (Low et al., 2015). In a review investigating the effectiveness of intergenerational programme participation for long-term care residents with dementia, Lu et al. reported that such participation ‘significantly increased pleasure levels’ among residents living with dementia (Lu et al., 2021, p. 9). Older participants have also been observed exhibiting physical signs of enjoyment during participation, such as ‘leaning in and smiling’ when children approached (Di Bona et al., 2019, p. 1686). However, further evidence regarding the strength and longevity of relationships following intergenerational participation is required. Stakeholders in Scotland perceived the enjoyment of older participants to be temporal in nature, with the potential to be affected by the progression of dementia through time.

Engagement was observed by interviewees as an outcome arising from programmes in the Scottish context and has been one of the most evaluated outcomes of intergenerational programmes involving people living with dementia. Findings that intergenerational activities can have a positive effect on engagement for older participants have been reported in several studies (Baker et al., 2017; Lee et al., 2007; Low et al., 2015 ). However, Galbraith et al. found that social engagement and activity engagement had been studied frequently, however, findings were mixed and ‘improvement was not universal’ (Galbraith et al., 2015, p. 372). Lu et al. found that intergenerational participation significantly reduced behavioural disengagement among long-term care residents living with dementia, but did not significantly improve active engagement, self-engagement, or passive engagement levels (Lu et al., 2021). Due to the mixed nature of the evidence, further studies investigating engagement outcomes for older participants would strengthen the evidence base.

Findings related to the wellbeing of older participants following participation in programmes are mixed and reflect the finding from the Scottish context that wellbeing outcomes can be difficult to attribute within intergenerational practice. There is evidence to suggest that wellbeing can be maintained among older participants living with dementia throughout the duration of programmes (Chung, 2009; Lim et al., 2019 ). In relation to the effects of intergenerational programmes on the physical health of older adults in general, Park suggested that although programmes cannot stop ‘the natural ageing process’ nevertheless ‘they [could] play an important role in delaying the speed of the rapid progression of degenerative/chronic diseases’ (Park, 2014, p. 4).

Lokon et al. (2018) found that the higher staffing ratio implemented during the intergenerational ‘Opening Minds Through Art’ programme resulted in higher overall intensity and frequency of wellbeing for people living with dementia (Lokon et al., 2019). In regard to a programme implemented in a hospital setting, McGeorge et al. observed that intergenerational workshops often facilitated physical movement for older patients, for example, by encouraging them to get out of bed (McGeorge et al., 2021). Skropeta et al. investigated intergenerational playgroups in aged care, and found, in contrast, a significant decline on an energy/fatigue subscale. The authors acknowledged this finding was suggestive of deterioration (Skropeta et al., 2014).

## Strengths and limitations

This study has several limitations that should be considered. The sample size of thirteen interviewees may be considered small, however, data saturation was met within the analysis (Braun & Clarke, 2021, p. 202). Interviews for this research were conducted remotely using computer-mediated communication due to pandemic restrictions, which may have affected data collection (Nadler, 2020). Interviews were limited to the Scottish context, however, this reflected the scope of the study, and contributes Scottish-specific findings that can be incorporated within comparative research. Gerritzen et al. recommended that further research seek to ‘explore the possible influence of the social-cultural setting’ through exploring programmes across different countries (Gerritzen et al., 2020, p. 242).

## Research implications

The contextual factors, mechanisms and outcomes identified in this study accord with evidence generated in studies internationally, though it is important to recognise that there are mixed findings in the evidence base and many gaps still to be addressed. Further empirical research into the perspectives, preferences, and experiences of people living with dementia in regard to intergenerational activities should be undertaken to better inform future programmes. As Weeks et al. observed in relation to nursing home settings, consultation with people living with dementia in the development of intergenerational programming is often lacking (Weeks et al., 2020). Research investigating the strength and longevity of the intergenerational relationships formed during programmes would be beneficial, along with further enquiry into how gender, race, ethnicity, and socioeconomic status may influence the experiences of older participants during programmes and the relationship-building process (Galbraith et al., 2015). Finally, research comparing programme implementation, stakeholder perspectives, and outcomes across different settings, for example, variations between care homes and community-based services, would be insightful.

## Conclusion

The implementation of intergenerational best practice through activity-based programmes appears to offer the potential to realise beneficial outcomes for people living with dementia. The perspectives of Scottish stakeholders were broadly supportive of policy aspirations within Scotland to tackle loneliness for people living with dementia alongside enhancing intergenerational dialogue between all age groups. This research contributes findings generated from the Scottish context that suggest particular contextual factors that can be addressed and mechanisms that can be implemented by practitioners involved in developing intergenerational programmes for people living with dementia.
